# Klebsiella aerogenes Causing Mitral Valve Infective Endocarditis With Multiple Septic Emboli to the Brain: A Case Report

**DOI:** 10.7759/cureus.97908

**Published:** 2025-11-27

**Authors:** Saif M Srouji, Mina Handal, Sandesh Lamichhane, Israel Martinez, Muhammad U Bakhsh

**Affiliations:** 1 Internal Medicine, Saint Agnes Medical Center, Fresno, USA; 2 Cardiology, Saint Agnes Medical Center, Fresno, USA

**Keywords:** adult cardiology, bloodstream infections, cardiovascular disease, cerebral septic emboli, infectious disease, infective endocarditis, internal medicine, klebsiella areogenes, native mitral valve endocarditis, septic embolic stroke

## Abstract

*Klebsiella aerogenes* is a Gram-negative bacterium commonly associated with urinary tract infections (UTIs); however, it can occasionally cause bloodstream infections and, rarely, infective endocarditis (IE). Severe complications related to *K. aerogenes* bloodstream infections, such as systemic embolization, have been described in the literature. In this report, we describe a case of *K. aerogenes* IE in an elderly male patient who initially presented to urgent care with a UTI. He had been diagnosed and prescribed a course of oral antibiotics; however, the treatment failed due to antimicrobial resistance. This led to the development of bacteremia and sepsis requiring hospital admission. He later developed endocarditis as confirmed by echocardiography, which was complicated by mitral regurgitation, respiratory failure, and multiple embolic central nervous system (CNS) strokes. He was treated with intravenous (IV) antibiotics and assessed by cardiac surgery, though operative management was deferred until his overall condition improved. This case underscores the importance of early recognition and timely management of IE in patients with persistent *Klebsiella* bacteremia unresponsive to initial therapy, emphasizing a multidisciplinary approach to achieve optimal outcomes, especially when dealing with such rare causative organisms.

## Introduction

Infective endocarditis (IE) is an infection of the endocardial surface of the heart that may involve the cardiac valves (native or prosthetic), mural endocardium, septal defects, or indwelling cardiac devices. It can present in either acute or subacute form, with the acute type often progressing rapidly and leading to systemic complications such as septic embolization [1].

The incidence of IE is estimated at 13.8 cases per 100,000 person-years, rising to nearly 500 per 100,000 person-years among individuals with predisposing risk factors [1]. Despite advances in diagnosis and management, in-hospital mortality remains between 20% and 30% [2-4]. *Staphylococcus*, *Streptococcus*, and *Enterococcus* species account for nearly 80% of cases [2]. Less common etiologies include Gram-negative organisms such as the HACEK group (*Haemophilus*, *Aggregatibacter*, *Cardiobacterium*, *Eikenella*, and *Kingella*), *Escherichia coli*, *Pseudomonas*, and *Klebsiella* species. In recent years, non-HACEK Gram-negative bacilli have shown a modest increase in incidence, particularly among elderly patients, those with multiple comorbidities, or those with implanted cardiac devices [5,6]. Nevertheless, infections caused by non-HACEK Gram-negative bacteria remain uncommon, representing less than 2% of all IE cases [1].

Within this group, *Klebsiella* species account for approximately 1% of native valve and up to 4% of prosthetic valve endocarditis [7]. In a review of *Klebsiella* endocarditis, prosthetic valve involvement was reported in 16.4% of cases, with *Klebsiella pneumoniae* identified as the most frequent pathogen. The clinical cure rate was 80.3%, while overall mortality reached 19.4%, which should alert clinicians when treating such infections to avoid delays in management [8]. Specifically, pathogenesis of *Klebsiella* in IE is by means of bacterial adherence to damaged endocardium through use of its adhesins, in addition to its ability for biofilm formation [7].

We present a case of *Klebsiella aerogenes* endocarditis involving the native mitral valve in an elderly male patient, complicated by septic shock, mitral regurgitation, and multiple systemic embolic strokes. This report aims to contribute to the limited literature on *K. aerogenes *IE and highlight the importance of early recognition and multidisciplinary management, given its potential for severe complications and high mortality.

## Case presentation

A 76-year-old male patient with a past medical history of lower limb deep venous thrombosis (DVT), hyperlipidemia, primary hypertension and chronic heart failure with preserved ejection fraction, presented to the emergency department complaining of shortness of breath that had started a few hours prior. He mentioned he had been seen at urgent care four days prior for complaints of fever and left flank pain, and that he was told he had a UTI. He was given a dose of Ceftriaxone 1G intravenously (IV) and subsequently sent home on a course of oral Cephalexin that he was taking up until the day of presentation. He said that he lives with his family who provides assistance with activities of daily living.

On initial examination, he appeared mildly distressed and had an SpO_2_ of 86% on room air thus he was placed on 6L oxygen by means of a nasal cannula, which improved his SpO_2_ to 94%. He was slightly tachycardic at a heart rate of 108 beats/minute. His blood pressure and temperature were normal. It was also noted that he had bilateral lower lobe inspiratory crackles on auscultation, 2+ lower limb pitting edema, cool extremities with mottled skin appearance; however, he stated that his legs appeared normal compared to baseline.

A quick chart review revealed that he had a urinalysis sent during the urgent care visit, and results were as presented in Table 1, in addition to urine cultures, which were know showing growth of *K. aerogenes* almost 96 hours later.

**Table 1:** Urinalysis results from urgent care visit Results of urinalysis obtained during the patient's visit to urgent care, 4 days prior to his hospital presentation, consistent with an active urinary tract infection.

Parameter	Value	Reference Range
Nitrites	Positive	Negative
Leukocyte Esterase	Positive	Negative
Bacteria	Many Present	None
Casts	White Blood Cell Casts	None

Initial lab tests during the current presentation were notable for parameters shown in Table 2.

**Table 2:** Results of the initial lab testing obtained on admission Results of the initial lab test obtained on admission, most notable for acute kidney injury and thrombocytopenia.

Parameter	Value	Reference Range
Sodium	133 mEq/L	135-145 mEq/L
Potassium	3.2 mEq/L	3.5-5 mEq/L
Chloride	96 mEq/L	96-106 mEq/L
CO_2_	25 mEq/L	22-28 mEq/L
Anion gap	12 mEq/L	8-12 mEq/L
Glucose	155 mg/dL	70-99 mg/dL
Blood urea nitrogen (BUN)	48 mg/dL	7-20 mg/dL
Creatinine	1.35 mg/dL	0.6-1.2 mg/dL
Glomerular filtration rate (GFR)	54 mL/min/1.73 m²	>90 mL/min/1.73 m²
Magnesium	2.3 mg/dL	1.7-2.4 mg/dL
Brain natriuretic peptide	140 pg/mL	<100 pg/mL
High-sensitivity troponin	14 ng/L	<20 ng/L
White blood cells (WBCs)	10.3 K/µL (84% Neutrophils)	4-11 K/µL
Red blood cells (RBCs)	4.6×10⁶/µL	4.7-6.1 × 10⁶/µL
Hemoglobin	14.3 g/dL	13.5-17.5 g/dL
Platelets	67 K/µL	150-450 K/µL

A quick bedside ultrasound done in the emergency department showed Kerley B lines in both lung fields, which corroborated with the findings from a chest X-ray image that showed bilateral pulmonary edema. A CT pulmonary angiography excluded the presence of pulmonary embolism but demonstrated the presence of bilateral pulmonary edema. The patient was admitted to the internal medicine service under the impression of acute hypoxic respiratory failure secondary to acute heart failure and unresolving UTI. He was started on IV Furosemide 40mg twice daily, and Ceftriaxone 1G IV daily. The following day, a transthoracic echocardiogram (TTE) was performed and revealed a hyperdynamic heart with left ventricular ejection fraction >70%, impaired left ventricular relaxation, heavily calcified aortic valve with severe aortic stenosis, peak velocity of 4.4 m/s and a mean gradient 47 mmHg. No pericardial effusion or valvular abnormalities were noted in this study. On day three of admission, peripheral blood cultures that were drawn on admission showed an initial growth of Gram-negative rods; later that day, a rapid response was called as the patient had a fever of 38.5, became hypotensive and now had altered mental status. The intensive care team was consulted and the patient was admitted to the intensive care unit (ICU) for septic shock. A central line was placed, and he started receiving Norepinephrine and Phenylephrine for blood pressure support, in addition to IV Meropenem 500 mg three times daily for broad Gram-negative bacterial coverage based on the infectious disease (ID) team recommendations.

**Figure 1:**
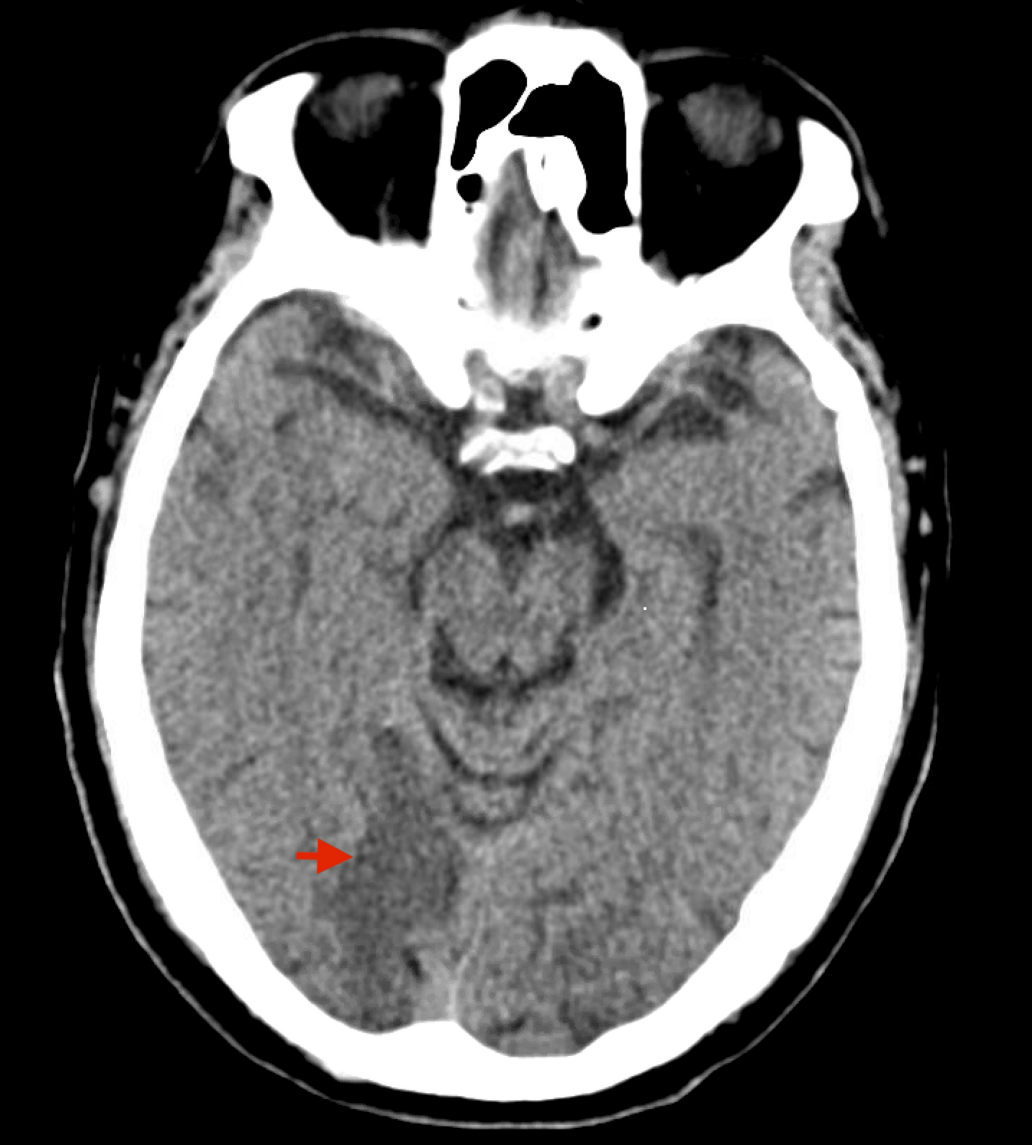
Head CT imaging Axial view of the CT scan of the head showing an area of hypo density in the right occipital lobe (red arrow) with loss of differentiation of gray and white matter consistent with the diagnosis of an acute stroke.

On day four, he appeared to have developed acute weakness of the left upper extremity and dysarthria with interval worsening of his encephalopathy. Physical examination revealed a rapid irregular rhythm confirmed to be atrial fibrillation with rapid ventricular response on electrocardiogram, and a new systolic murmur, which raised clinical suspicion for infective endocarditis and septic emboli to the brain. A head CT scan showed a hypodense area involving the right occipital lobe, suggestive of an acute infarct in the right posterior cerebral artery (PCA) distribution with loss of differentiation of gray and white matter, which was confirmed by a CTA head showing occlusion of the distal PCA (Figure 1).

**Figure 2:**
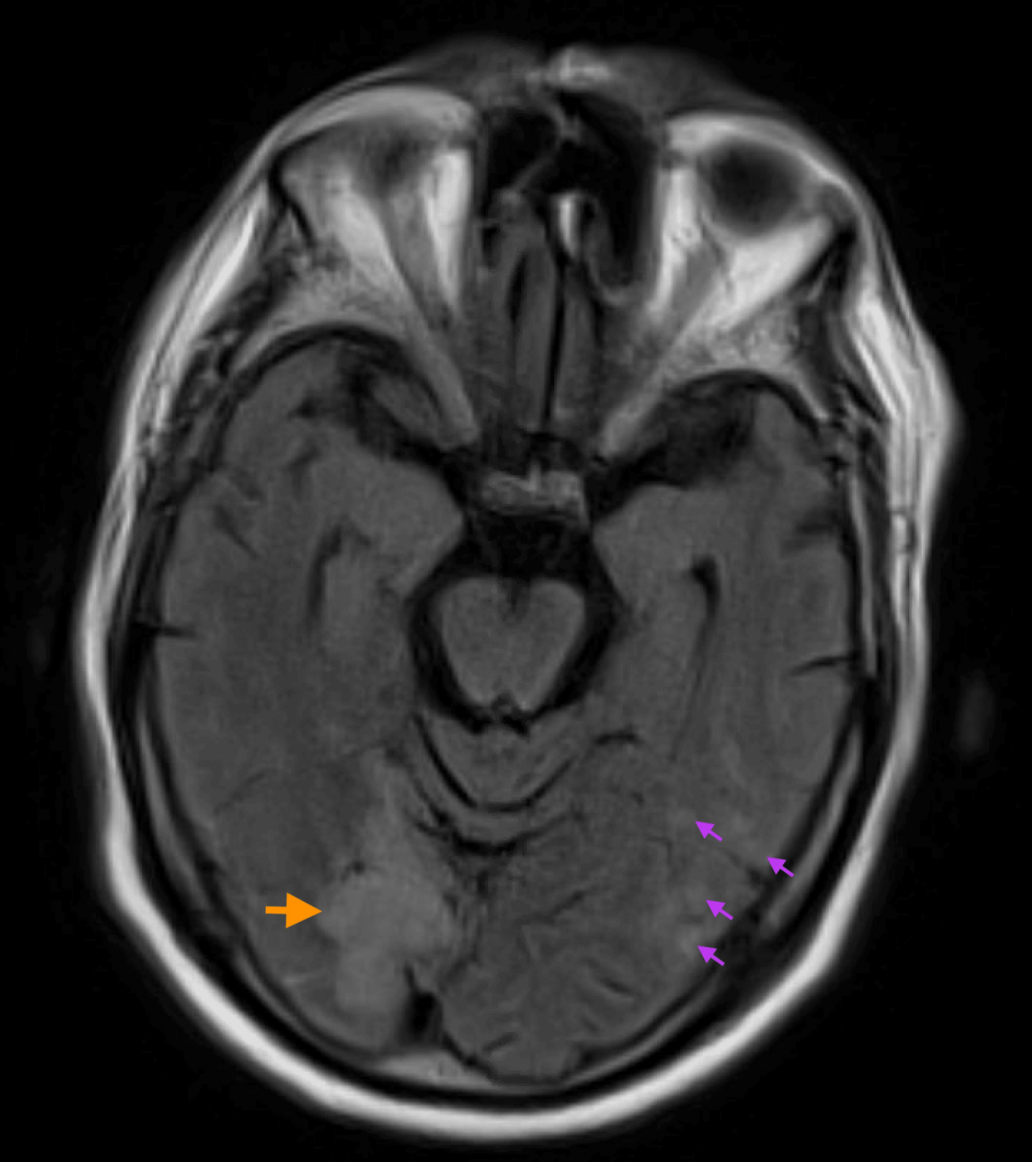
Brain MRI imaging Axial view of the brain MRI showing an area of hyperintensity in in the right occipital lobe (orange arrow) consistent with the finding on the Head CT, in addition to numerous small areas of hyperintensity (purple arrows) signifying acute and subacute infarcts.

He was not a candidate for tenecteplase (TNK) as there was no large vessel occlusion. He was started on a heparin infusion for atrial fibrillation anticoagulation, in addition to Amiodarone infusion for rhythm and rate control. A subsequent brain MRI without and with contrast showed a small volume of subarachnoid hemorrhage within the left central sulcus in addition to numerous acute and subacute infarcts in areas of distribution of the anterior and posterior circulation, raising concern for cardiac stroke etiology (Figure 2). Heparin infusion was stopped after noticing the subarachnoid hemorrhage based on consultation with the Neurology service.

On day five, blood cultures had finalized and revealed growth of *K. aerogenes* with the following antimicrobial susceptibility profile (Table 3).

**Table 3:** Results of the antibiotic sensitivity testing for blood cultures that grew Klebsiella aerogenes Results of the antibiotic sensitivity testing showing most importantly, that* K. aerogenes* that was isolated from blood cultures was resistant to Ceftriaxone, which is the likely reason of initial UTI treatment failure.

Agent	Result
Aztreonam	Resistant
Cefepime	Susceptible
Ceftriaxone	Resistant
Ciprofloxacin	Susceptible
Gentamicin	Susceptible
Levofloxacin	Susceptible
Meropenem	Susceptible
Piperacillin/Tazobactam	Intermediate
Trimethoprim/Sulfamethoxazole	Susceptible

A transesophageal echocardiogram (TEE) was performed on day five and showed the presence of a 5-mm vegetation on the posterior leaflet of the mitral valve, with moderate mitral regurgitation, heavily calcified aortic valve with severe aortic stenosis, and no vegetations were seen on any of the other valves (Video 1). This finding confirmed the diagnosis of definite infective endocarditis according to the Duke criteria.

**Video 1:** Transesophageal Echocardiogram Transesophageal echocardiogram (TEE) imaging showing presence of vegetation on the posterior leaflet of the mitral valve in addition to a doppler flow view showing mitral regurgitation, this was confirmation for diagnosis of infective endocarditis in the setting of bacteremia and multiple septic systemic emboli.

Repeat blood cultures from day five did not show any bacterial growth. The following day, the cardiothoracic surgery team evaluated the patient again and recommended he be transferred to a higher level care center for surgical replacement of the aortic and mitral valves once he is medically stable. The ID team also made the same recommendation and adjusted the antibiotics by adding IV Gentamicin to IV Meropenem for a synergistic effect.

On day nine of admission, vasopressors had been weaned off, and he was transferred to the step-down unit and to the medical floor afterwards. The ID team recommended continuation of IV antibiotic treatment for a total duration of at least six weeks before re-evaluation for possible extension of treatment. He remained on the internal medicine service, receiving antibiotics and rehabilitative physical therapy until he was transferred out. He had been sent home without undergoing surgery at that time, because the cardiothoracic surgery team believed that his surgery could be planned for a later date once he had completed physical rehabilitation for his stroke and finished his course of IV antibiotics. A follow-up TEE approximately eight weeks later did not show any residual vegetations affecting the mitral valve. He still follows with the cardiothoracic surgery team; however, he does not appear to have undergone the surgery yet due to the high risk associated with the surgery, considering his poor functional status.

## Discussion

Gram-positive cocci (such as *Staphylococcus*, *Streptococcus*, and *Enterococcus*) predominate in IE because they adhere well to damaged endocardium (via adhesins and platelet-fibrin matrices) and are usually found on skin and oral mucosa (sites that commonly seed bacteremia) [9,10]. *Klebsiella* species are an uncommon cause of IE, representing only a small fraction of all reported cases. While non-HACEK Gram-negative bacilli account for roughly 1.5%-10% of IE overall, *Klebsiella* infections constitute less than 2% of this subset, most commonly involving *K. pneumoniae* or *K. oxytoca* [2,5,8]. Recent data reaffirm that *Klebsiella* endocarditis remains rare but clinically significant, most often associated with healthcare exposure or invasive procedures [5,6]. Pathogenesis involves bacterial adherence to damaged endocardium or prosthetic material, aided by fimbrial adhesins and biofilm formation, which promote persistence and protect against host immune defenses [7]. Predisposing factors include advanced age, diabetes mellitus, renal insufficiency, malignancy, intravenous catheters, and prosthetic valves [3,6]. Community-acquired cases remain exceedingly rare, and their occurrence, particularly in immunocompetent patients, emphasizes the importance of maintaining diagnostic consideration when Gram-negative bacteremia persists without an identifiable source.

Diagnosing *Klebsiella* IE can be challenging, as Gram-negative bacteremia rarely results in endocarditis. Consequently, diagnostic evaluation such as echocardiography is often delayed until complications arise, including persistent bacteremia unresponsive to therapy, new cardiac murmurs or peripheral signs of IE, or systemic embolic events, as in our patient who developed multiple embolic strokes prior to diagnosis. TEE remains the most sensitive imaging modality, capable of identifying vegetations or abscesses that may not be detected on transthoracic echocardiography [1].

In a recent retrospective cohort study, complications occurred in 74% of patients with non-HACEK Gram-negative infective endocarditis, most frequently heart failure, embolic events, and abscess formation [11]. In a systematic review of 67 reported cases of *Klebsiella* endocarditis, Ioannou et al. (2021) reported an overall mortality rate of 19.4% [8]. These findings are consistent with those of Bouza et al. (2021), who reported that non-HACEK Gram-negative infective endocarditis carries a high mortality rate of approximately 20%-30% and frequent complications despite appropriate therapy [5].

Management of *Klebsiella*-infective endocarditis remains challenging due to both intrinsic and acquired antimicrobial resistance. *Klebsiella* species commonly produce β-lactamases, conferring natural resistance to ampicillin and sometimes to extended-spectrum β-lactams through extended-spectrum β-lactamase (ESBL) and carbapenemase production [12]. Treatment generally follows recommendations for non-HACEK Gram-negative IE, emphasizing a minimum six-week course of intravenous combination therapy, typically a β-lactam/β-lactamase inhibitor or carbapenem with an aminoglycoside or fluoroquinolone, guided by susceptibility testing [1]. Early surgical consultation is recommended, especially in cases complicated by heart failure, persistent bacteremia, prosthetic valve involvement, or large vegetations [1]. In a recent Brazilian multicenter cohort, 42% of non-HACEK Gram-negative IE patients underwent surgery, highlighting its frequent necessity [6]. Surgical decisions should balance operative risk with the poor outcomes associated with medical therapy alone.

Our patient’s presentation is consistent with previously reported cases of *Klebsiella* endocarditis, which typically involve native left-sided valves and occur in older or comorbid patients [7,8]. The infection in this case affected the native mitral valve, presenting with fever and a new murmur, complicated by multiple embolic strokes, causing the patient to exhibit neurological deficits prior to obtaining cardiac imaging, leading to the recognition of endocarditis. The predisposing factors were advanced age and prior healthcare exposure in a skilled nursing facility, with a recently diagnosed urinary tract infection later confirmed as the likely source. The organism exhibited resistance to third-generation cephalosporins, requiring treatment with meropenem and gentamicin, consistent with recommendations for extended-spectrum β-lactamase (ESBL)-producing strains [1,3,5]. Blood cultures cleared rapidly after initiating therapy, and the patient was managed medically without immediate surgery. Delaying the surgery was made after a risk assessment considering the relatively moderate size of the vegetation, early clearance of bacteremia with antimicrobial therapy, and overall physical condition of the patient.

In summary, *Klebsiella* endocarditis is a rare but aggressive infection associated with significant morbidity and mortality. Despite advances in antimicrobial therapy and supportive care, outcomes remain poor when diagnosis is delayed. This case contributes to the growing literature on *Klebsiella* IE by illustrating native mitral valve involvement complicated by multiple embolic strokes in a patient without typical urinary tract risk factors.

## Conclusions

*K. aerogenes* infective endocarditis is an uncommon but severe clinical entity, typically affecting elderly or comorbid patients and often associated with prior healthcare exposure or urinary infection. It can progress rapidly, leading to complications such as embolic stroke, heart failure, and multi-organ dysfunction. This case emphasizes the importance of maintaining diagnostic consideration for infective endocarditis in patients with persistent Gram-negative bacteremia, even when an alternative reason for persistent bacteremia, such as a resistant urinary infection, appears more likely. This case demonstrates that early echocardiographic evaluation and diagnosis, timely escalation to targeted combination antimicrobial therapy, and coordination between infectious disease, cardiology, and critical care teams are essential for modern multidisciplinary team management. Despite this, outcomes can be unfavorable due to the severity and virulence of Gram-negative bacterial endocarditis. Although our patient experienced a significant setback, suffering multiple strokes that require prolonged antimicrobial therapy and physical rehabilitation, he continues to attend cardiothoracic surgery evaluation for planned operative intervention. We aim to add to the limited literature on *Klebsiella* endocarditis involving native valves complicated by multiple embolic strokes, by showcasing antimicrobial resistance, diagnostic challenges and "endocarditis team" management in accordance with the most recent guidelines.
